# Applying Deep Learning in the Training of Communication Design Talents Under University-Industrial Research Collaboration

**DOI:** 10.3389/fpsyg.2021.742172

**Published:** 2021-12-15

**Authors:** Rui Zhou, Zhihua He, Xiaobiao Lu, Ying Gao

**Affiliations:** ^1^School of Textile Engineering and Art, Anhui Agricultural University, Hefei, China; ^2^Art and Design College, Zhejiang Gongshang University, Hangzhou, China

**Keywords:** deep learning, RNN, higher vocational computer science specialty, talent cultivation, fused attention model, communication design courses

## Abstract

The purpose of the study was to solve the problem of the mismatching between the supply and demand of the talents that universities provide for society, whose major is communication design. The correlations between social post demand and university cultivation, as well as between social post demand and the demand indexes of enterprises for posts, are explored under the guidance of University-Industrial Research Collaboration. The backpropagation neural network (BPNN) is used, and the advantages of the Seasonal Autoregressive Integrated Moving Average model (SARIMA) model are combined to design the SARIMA-BPNN (SARIMA-BP) model after the relevant parameters are adjusted. Through the experimental analysis, it is found that the error of the root mean square of the designed SARIMA-BP model in post prediction is 7.523 and that of the BPNN model is 16.122. The effect of the prediction model that was designed based on deep learning is smaller than that of the previous model based on the neural network, and it can predict future posts more accurately for colleges and universities. Guided by the “University-Industrial Research Collaboration,” students will have more practice in the teaching process in response to social needs. “University-Industrial Research Collaboration” guides the teaching direction for communication design majors and can help to cultivate communication design talents who are competent for the post provided.

## Introduction

At present, the major of communication design is popular in higher education, and the demand for high-quality talents required by enterprises is relatively high. However, unsuitable teaching methods and teaching contents may not match the supply and demand in some situations. The actual employment rate is not particularly ideal, and the talent output cannot meet the real demand (Nashold and Krishnan, 2020). In this case, the concept of “University-Industrial Research Collaboration” brings new vitality to this phenomenon. The principle of this concept requires cooperation among various elements and interaction among the four sectors, namely, society, university, research, and practice. In the teaching stage, universities can use other environments such as social enterprises as the basis of the teaching environment and transform knowledge dissemination into a more direct teaching method, which greatly improves society-required skills and abilities. In this process, it is necessary to find an algorithm model that can analyze talent ability and provide a theoretical basis for this concept (Msosa, 2020).

Related institutions and some scholars have studied the matching degree of talent quality and enterprise demand using the traditional questionnaire and statistical analysis, and there are few relevant studies on it from the perspective of data mining. Scholars use logistic regression and decision tree algorithms to build a multidimensional occupation evaluation model, and the university can use it to analyze the professionalization and personalization of students and to predict their professional quality, improving the employment rate. Based on data mining and the data analysis algorithm, the logistic regression and decision tree algorithm of the multidimensional occupation evaluation model are proposed (Wagner-Muns et al., 2017), and they can predict the ability of college graduates and meet the common needs of the professionalization and personalization of students, improving the employment rate (Chattha et al., 2019). Scholars also used the network to obtain the employment information of past graduates and to use the support vector machine regression model to predict the unemployment rate and overcome the shortcomings of previous data statistics. Later, they used the clustering dimension reduction and the classification of urban-related demand and adopted the relevant model to predict the talent demand in the region in a certain period. Also, based on the perspectives of industrial discipline and industrial structure, some people used a large number of data as the sample support to design the relevant prediction model through the partial least squares regression method. Based on this, the big data crawler technology is used to obtain information on employment recommendations, and then, the mixed recommendation platform is constructed, and its prediction rate for employment is more accurate. Its shortcomings are as follows: (1) the research methods of modeling and quantifying the employment data are few; (2) the time, the accuracy of matching, and the utilization rate are not high; and (3) the relevant models have much room for improvement.

Based on the Seasonal Autoregressive Integrated Moving Average model (SARIMA), a new SARIMA-backpropagation neural network (SARIMA-BP) prediction model is constructed by combining deep learning with the backpropagation neural network (BPNN), and its excellent mining time sequence non-linear law is used to optimize the model. The overall structure is as follows: First, the realization of “University-Industrial Research Collaboration” and its post prediction ability are discussed, and several traditional prediction methods are expounded. Second, the time sequence prediction method and the neural network prediction method are selected, and the advantages of these two methods are integrated, laying the foundation for the model designed in this study. After the prediction model is designed, its prediction results are compared with those of the neural network prediction model. The results show that the accuracy of the prediction model designed is more accurate, and it has an excellent performance in predicting future posts. It provides technical support for the realization of “University-Industrial Research Collaboration.” It enables enterprises, universities, students, practice, and research to connect in the teaching concept of “University-Industrial Research Collaboration.” In the cultivation of communication design talents, the teaching content that meets the needs of enterprises is obtained, so that students can have the ability required by social development and improve their competitiveness, which has great significance in the reform and development of communication design teaching.

## Literature Survey

Luo et al. (2018) argued that different industries have different demands for talents in the industry while different industries need to update themselves to meet the requirements of the times. Under the era of “big data,” the requirements for colleges and universities in talent training are high. As an educational institute, how to adapt to the trend of the era of “big data” and to provide more competitive talents for the society needs to be explored. Also, the integration of colleges and enterprises is the problem that colleges and universities need to face. As the main training institution of talents, colleges should make full use of relevant resources and change the teaching methods according to social needs and major characteristics. This proves that colleges in many countries have conducted relevant research on major settings to match the post requirements. Arrafii (2021) conducted a survey on the career evaluation of college graduates by taking the product of evaluation results of different dimensions and the weight of the evaluation dimension as the score of students, and the score reflects the matching degree between the ability of students and their posts. Mahmut and Suna (2020) conducted a questionnaire survey on the matching of the majors of students in colleges and the first posts of college graduates by an experiment on the matching of the posts of students in recent years under multiple dimensions. They concluded that the matching degree of the posts of students and their majors in colleges is low, and there is a negative correlation between the two. Also, frequent job changes can reduce the work efficiency of and increase the working pressure of students.

In terms of the optimization of the methods, scholars in related fields have their own different methods. Awujoola et al. (2021) used data mining and data analysis algorithms to design a multidimensional career evaluation model based on the logistic regression and decision tree algorithm. College graduates can be provided with professional quality evaluation combined with personal information using this evaluation model to improve the employment quality of college graduates. The effect of the improved time prediction model is more accurate than that of the single model. Livieris et al. (2019) established a prediction model for graduate employment by improving the semi-supervised classification method. After a lot of iterative training, it is found that the improved algorithm is superior to the traditional algorithm. Pramanik et al. (2021) predicted that the number of educational institutions is carried out based on the traditional autoregressive integrated moving average (ARIMA) model and the neural network, which provides a method model for analyzing industry development. Scholars use the previous talent demand sequence for clustering dimension reduction based on the fitting prediction of the abovementioned ARIMA model, and the demand for the professional market is obtained.

Based on the post demand theory and prediction model, the direction for the cultivation of talents in colleges and universities is provided. The research has certain practical significance.

## Research Model

### Forecast Method of Professional Employment Demand

**FIGURE 1:**
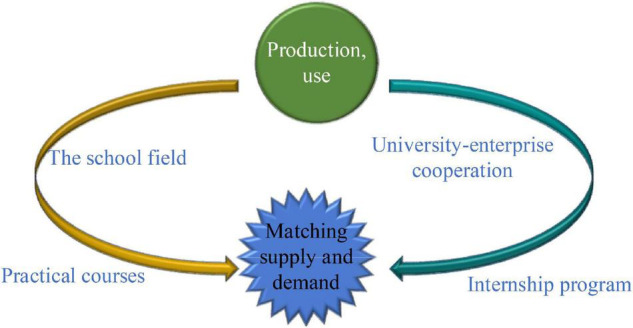
Design of the ideal curriculum.

In professional talent education, the ideal “University-Industrial Research Collaboration” is difficult, and its ideal curriculum needs to be designed by connecting universities and enterprises, as shown in Figure 1.

**FIGURE 2:**
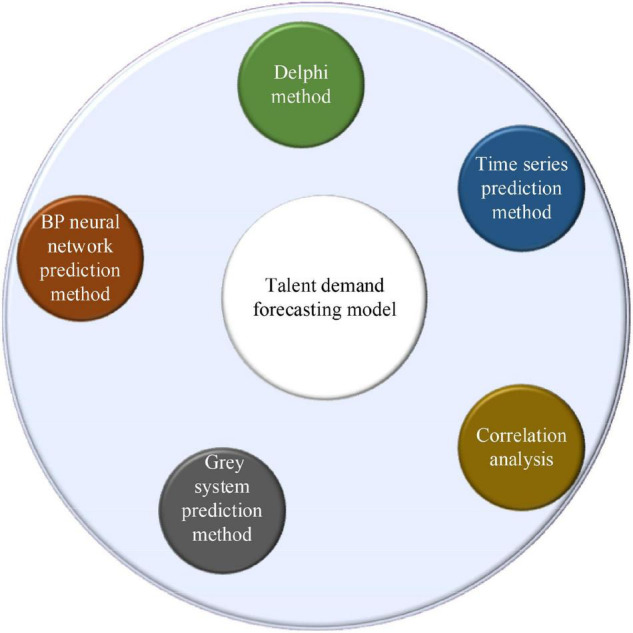
Talent demand forecasting model.

Since it emphasizes the relevance of the university, enterprise, and practice, it is a key to improve the matching degree of talent ability and enterprise demand. Because it has a close relationship with the enterprise post demand, in other words, the enterprise post demand should be the basis of its curriculum. In the design of the curriculum, there may have many problems. Also, a good prediction of enterprise post demand is needed. In terms of professional demand forecast, the research group changes from the government to scholars, and the forecast methods they use are continuously developed and improved through exploration, practice, and innovation. Through its research methods, the previous professional demand forecast methods are shown in Figure 2.

**FIGURE 3:**
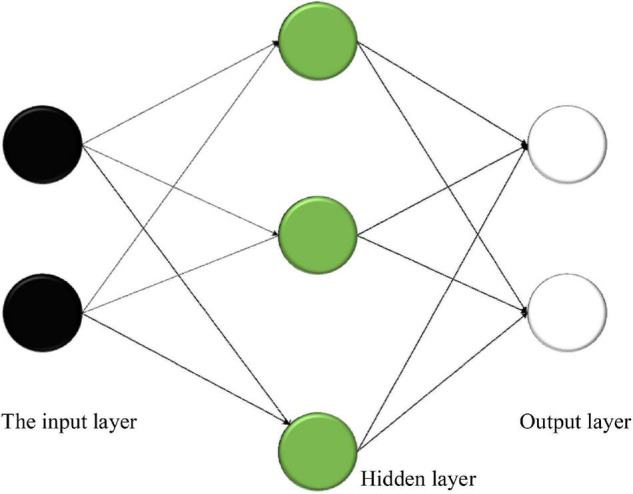
Structure of neurons.

(1) Delphi method. It can also be called the expert investigation method, which shows that authoritative scholars analyze and forecast the talent ability required by the talent market of enterprises. The essence of forecast means is to clarify private opinions in real time. Through a certain forecast process, the method of non-interference is adopted, and the private opinions of scholars on the demand for enterprise talents are predicted.
  In general, the use of the Delphi method needs to clarify the members of the expert group, which requires the team members to have a certain professional ability. Then, they need to work with investigators. The size of the group should be more than 10, and the investigation question needs to be simple, clear, and convenient to answer before the investigation. The knowledge that the scholars need to use should be prepared, and the relevant information should be distributed to each person in the group in the first forecast. It is unnecessary to pay more attention to the requirements for answering the questions and the recovery time, or it has a bad impact on the recovery rate. After the repeated implementation of the abovementioned process is conducted, the second round starts. After all the scholars complete the task, the subjective views adjust until the differences in their views are the smallest. Finally, the conclusions are drawn according to the statistics and analysis (Khan and Gupta, 2020).
(2) Time sequence prediction method. Time sequence generally refers to the numerical group in which the values and actual values of relevant variables are arranged in chronological order with a certain interval in the economic activities of the first pass. The forecast method is to analyze the law of the object in a certain period by arranging the previous state and time of the research object. According to the law, the demand for social talents is predicted. In other words, if the predicted object is compared to a time function, the time feature is also measured, and the relationship between the related object and time is analyzed, completing the prediction, collecting the time axis information of the function, establishing and absorbing the relevant function, and carrying out the relevant forecast analysis. All kinds of changes in time have a certain impact on the results. Most of the previous time sequence prediction methods are based on the relevant parameters after the relevant models are implemented. With the continuous development of time sequence forecast methods, deep learning and dynamic parameter forecast models also have good results (Wang Y. et al., 2018). For example, the complexity of the calculation is determined by the number of support vectors rather than the dimension of the sample space, thus avoiding the “curse of dimensionality.” The Bayesian network (BN) has advantages in processing small datasets. This is because Bayesian learning is an integrated learning method. It has a good effect on small samples and does not cause overfitting. Matrix factorization (MF) can map the high-dimensional matrix into the product of two low-dimensional matrices, which provides a way to solve the problem of data sparsity. It is easy to implement and has high prediction accuracy. The Gaussian process (GP) is excellent in dealing with complex regression problems such as high dimension, small samples, and non-linearity and has good generalization ability.
(3) Correlation analysis. It generally refers to the analysis of the talent market and its related environments related to the detailed indexes, the use of indexes of the previous data to seek the basic parameters required for the establishment of the model and the successful construction of the model, and then, the prediction is conducted (Jha et al., 2021). In this regard, the regression model is used in the prediction. The mathematical principle of the regression model is the square method, and the mathematical function is used to speculate the prediction results in reality. The regression analysis is based on determining the dependent variable, looking for the variables that have a relationship with it before and after, and establishing the correlation function equation. At a certain time, the law of independent variables is used to predict the specific changes of future dependent variables. During the analysis, the number of independent variables is the key to divide the regression model. If there is only one independent variable in the relationship with the dependent variable, the regression prediction model is the one-variable regression prediction model. If the independent variable is not unique, it is the multiple regression prediction model (Alhnaity and Abbod, 2020).
(4) Gray system prediction method. The mathematical model is composed of a gray system called gray models (GMs), and the one-dimensional first-order gray prediction model is used more widely. The means of GM operation is to accumulate and generate the incomplete and lack of change in law-related information, slowly making the research object has a certain growth. On this basis, the differential equation model is established to achieve the purpose of prediction. The advantages of the gray system prediction method are as follows: there are not many initial data needed, and whether the data change or not has less effect on the prediction accuracy. The gray system prediction method is simple in structure and adjustment, and it is suitable for the short-term prediction. It can predict the change of objects. However, its disadvantage is that its prediction accuracy will decrease with the increase of unknown information in the system (Davis et al., 2019).
(5) BPNN prediction method. Based on the previous deep learning neural network model, a multilayer feedforward network model is implemented through the error backpropagation (BP) algorithm (Wang C. H. et al., 2018), which has three layers, namely, the input layer, the hidden layer, and the output layer. The details are shown in Figure 3.

**FIGURE 4:**
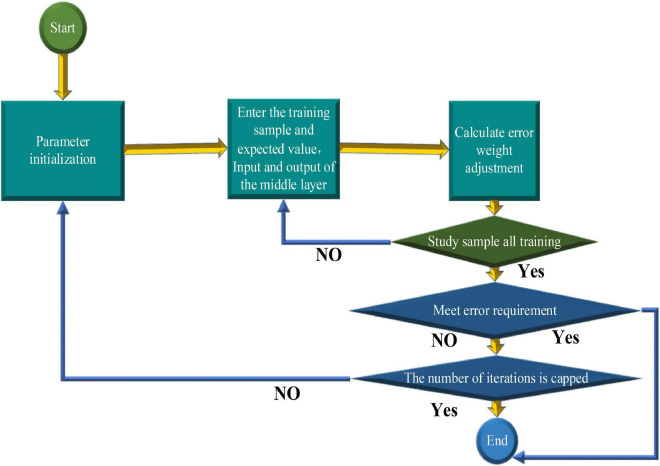
Flowchart of the BPNN algorithm.

The most critical steps in the modeling process are training and performance testing (Yang and Chen, 2019). The training should clarify the neural units of the input layer and the output layer and the operating parameters of the model. The flowchart of the BPNN algorithm is shown in Figure 4.

Logically speaking, the whole process of the BPNN is the complete propagation of information from entry to exit, and relevant optimization algorithms are used. The small adjustment of parameters can improve the accuracy of the model (Karasu et al., 2020). The BPNN prediction method has its unique features. It can make a good solution to the relevant non-linear phenomena or adjust the structure of the model in a certain sense. In other words, it can change the number of hidden layers and the number of neural units according to their own needs. In addition, the difference between the models will also change the performance, so that the model can be configured in different fields (Gates et al., 2017).

### Time Sequence Model

The time sequence is widely used in real life. It is used in the prediction of the changes of temperature, price, and the birth rate of the population, as well as the data source following certain rules (Song et al., 2020). The laws, trends, and the general situations of the changes can be found by using time sequence. Three points need to pay attention to in using time sequence, and they can be summarized as follows:

(1) The relevant data in the time sequence are inseparable from time, and the existence of time requires the existence of data. There is no strict requirement on whether there is a correlation function (Jiang et al., 2017).
(2) There may be some uncertainties in the data sequence. Although certain objective laws can be found from previous data, the law is artificially formulated, resulting in a certain deviation in the prediction. Nevertheless, it still provides a reference because this kind of uncertainty may also reflect certain laws (Tealab, 2018).
(3) There will be a correlation before and after a certain period. The summary of this correlation system is also the regularity of the sequence data. The time sequence model, whose mean and variance are not particularly large in a certain period, is called the stationary time sequence model, which does not have periodicity. The multiple linear regression models generally cover simple stationary time sequence autoregressive (AR), moving average (MA) model, ARIMA, and non-stationary time sequence ARIMA model.

In the process of practical application, the time sequence generally obtained is not the stationary time sequence in the above because it is difficult to analyze the data law. Some scholars put forward the ARIMA model to solve the conflict between practice and theories (Yao et al., 2020). It is also called the differential ARMA model. It is used in a single-variable environment, such as in the economic and meteorological prediction of the country, and has a strong ability. Although it has been developed in the field of education, the differential ARMA model has not made some achievements (Lu et al., 2018).

The Seasonal ARIMA model is the so-called SARIMA model, which is a special differential moving AR model. It adds seasonal elements to the conventional ARIMA model. The periodic parameter “s,” the periodic AR, the differential (I), and MA are used to reduce the prediction error of seasonal trends for ARIMA in the period. The Seasonal ARIMA model is written as SARIMA (p, d, q) (P, D, Q) s. P, D, and Q refer to the parameter values of seasonal changes, p, d, and q slow the seasonal parameter values, and “s” has a certain impact on P, D, and Q, representing the annual seasonal cycle. The month of the seasonal cycle model is 12. When the time sequence Y*_*t*_*, *t* = 1, 2… has the trend and periodicity, the stationary sequence of the first-order and the second-order seasonal difference sequence can be obtained after the D-th quarter difference, and the form of the model can be written as follows:


(1) $$\phi \,\left(B\right)\,{\left(1-{\text{B}}^{s}\right)}^{D}\,Y_{t}=\Theta \,\left(B\right)\,\varepsilon_{t}$$

In the above equation,


(2) $$\phi \,\left(B\right)=1-\phi_{1}\,B^{s}-\phi_{2}\,B^{2}\,s-\ldots \,\phi_{P}\,B^{\mathrm{Ps}}$$

(3) $$\Theta_{Q}\,\left(B\right)=1-\Theta_{1}\,{\left(B\right)}^{s}-\Theta_{2}\,{\left(B\right)}^{s}\,s-\ldots -\Theta_{P}\,\left(B\right)\,B^{\mathrm{Ps}}$$

ϕ(B) is the AR characteristic polynomial of time sequence, Θ(B) is the seasonal MA characteristic polynomial, ε_*t*_ is the residual, B is the delay operator, and P is the seasonal regression order. This model is generally expressed as follows:


(4) $$\varphi \,\left(B\right)\,\phi \,\left(B\right)\,{\left(1-\text{B}\right)}^{d}\,{\left(1-{\text{B}}^{s}\right)}^{D}\,Y_{t}=\theta \,\left(B\right)\,\Theta_{Q}\,\left(B\right)\,\varepsilon_{t}$$

In Equation 4, the correlation of the seasonal period in the sequence is mapped by θ(B) and Θ_*Q*_, and the quantitative relationship between the periods is mapped by φ(B) and ϕ(B). When (P, D, Q) is 0, the sequence has no seasonal characteristics. At this time, it cannot be called the SARIMA model but the ARIMA model (Yoon et al., 2019).

The SARIMA model is widely used in real life, and it can be used in transportation, hospitals, and many other important fields. The cultivation of communication design majors needs to know about social recruitment prediction methods. Some scholars use the ARIMA model to make predictions and find that the recruitment data contain the characteristics of the trend and seasonal fluctuations. Obviously, this does not have a very direct relationship with the research topic because the prediction does not apply the SARIMA model to specific jobs, which does not directly promote the direction of talent training and practice. This model has good stability and performance and overcomes the shortcoming of the ARIMA model by adding seasonal elements to the specific post prediction.

### Implementation of the SARIMA-BPNN Model

**FIGURE 5:**
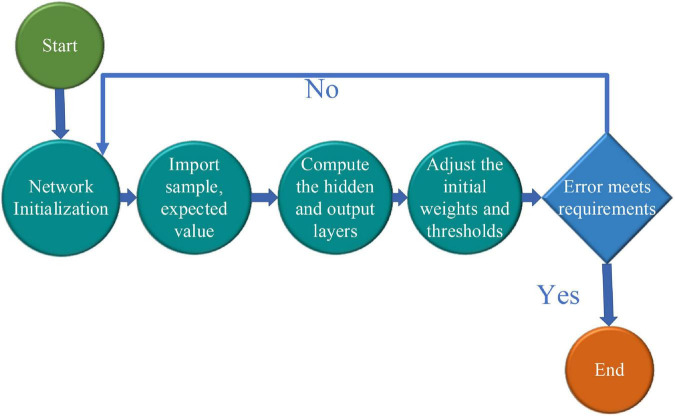
Learning and training of the three-layer BPNN model.

In the complex environment of society, the specific post demand sequence is impossible to be interfered with only by a single factor. The related factors not only have linear laws but also have non-linear laws. The predicted objects of the time sequence prediction model can be regarded as linear functions related to variables. The SARIMA model alone may cause different errors. The neural network model can better mine the non-linear law in the time sequence, and scholars apply it to the post prediction in the future. Because a three-layer BPNN approximates a random rational function and the post prediction data are regular, the three-layer BPNN is used to establish the SARIMA-BPNN model to reduce errors (Liao et al., 2019). The learning and training of the three-layer BPNN model are shown in Figure 5.

Specifically, the learning and training of the three-layer BPNN model are explained as follows:

(1) Operation initialization. Sample *X* and the expected value *Y* are the inputs. The input sequence and the expected output sequence of the sample are denoted as (*X*, *Y*), and the endpoints of the network input layer and the output layer are denoted as *n* and *m*. When *i* = 1,2,3,4,…*n*, *j* = 1,2,3,4…,*l*, *k* = *j* = 1,2,3,4…,*m*, the number of the nodes of hidden layer *l* can be written as follows:

(5) $$l=\sqrt{m+n}+d,1\leq d\leq 10$$

(2) Connection of the weights and thresholds of each layer. The hidden layer is calculated through the input signal of the input layer, the hidden layer is denoted as H*_*j*_*, and the output layer is denoted as O*_*k*_*.

(3) According to the output layer O*_*k*_* and the expected output *Y*, the error e*_*k*_* is calculated, and the error value is used to define the initial weight at the threshold. The calculation equation e*_*k*_* is expressed as follows:

(6) $$e_{k}=\frac{1}{2}\,\sum_{k=1}^{m}{\left(Y_{k}-O_{k}\right)}^{2}$$

(4) Operation forward propagation and error inverse propagation. The global error *E* is obtained based on operation forward propagation and error inverse propagation to determine whether the learning is over. The calculation equation of *E* is expressed as follows:

(7) $$E=\frac{1}{2\,t}\,\sum_{t=1}^{m}\sum_{k=1}^{m}{\left(Y_{k}-O_{k}\right)}^{2}$$

**FIGURE 6:**
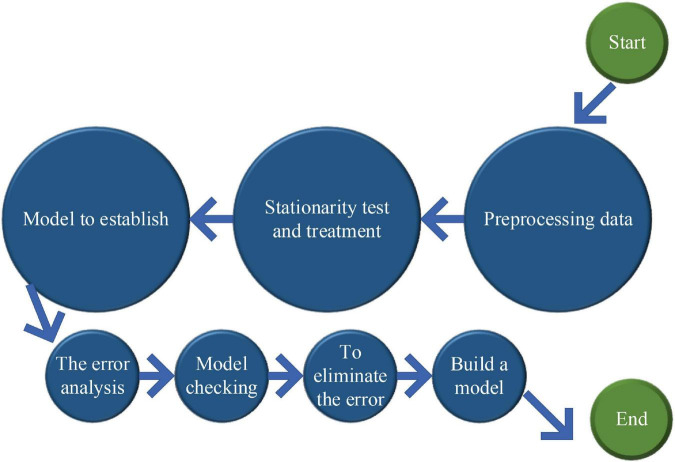
Prediction steps of the SARIMA-BPNN model.

Python is used to construct the neural network module. The network structure is adjusted to 4-8-1, the 1–4 order of the difference data of the residual sequence is used as the input variable, the residual sequence is used as the output variable, the hidden node is defined as 8, and the output node is 1 after the cost of infrastructure, multiple tests, and the error are considered. The final reason why the neural network can approximate all functions is that it adds a non-linear function as the incentive function. Therefore, the BPNN model is very important for the choice of incentive function. For the general incentive function, the complexity of the choice is relatively simple. Sigmoid, which is frequently used in this field, is used as an incentive function in this model. In general, the occurrence of gradient disappearance and convergence is also satisfactory. It is very suitable for the SARIMA-BPNN model to predict specific post data. The prediction steps of the SARIMA-BPNN model are shown in Figure 6.

The root mean square error (RMSE) is introduced as the evaluation index of post prediction to make the evaluation more rational and objective in evaluating whether the model is good or not. This index is very sensitive in calculating the error of a set of measured values and can well map the error between the data and the actual situation and the predicted value of the model. Its principle is that the square sum of the error between the fitting value and the real value negatively correlates with the square root ratio of the number of observations *n* and the accuracy. The equation is expressed as follows:


(8) $$R\,M\,S\,E=\sqrt{\frac{\sum_{i=1}^{n}{\left(X_{o\,b\,s,i}-X_{\mathrm{model},i}\right)}^{2}}{n}}$$

In Equation 8, *X*_*obs*,*i*_ is the original value, *X*_*model*,i_ is the predicted value, and *n* is the length of the data.

## Experimental Design and Performance Evaluation

### Experimental Environment

Based on Python, the post information in the 2020 popular recruitment website is crawled to make a clearer and objective evaluation by the SARIMA-BPNN model, and the specific demand posts of enterprises near the universities in the relevant regions are analyzed. The demand posts are predicted by the model, and the two sets of data are statistically summarized. The real situation of job demand is compared with the situation predicted by the model to understand the performance of the model in the specific environment. In placing the relevant data in the specified coordinates, it is more intuitive to know whether the gap between them is large and whether the model is reliable.

### Dataset Collection and Preprocessing

The new data available after the crawler are not complete and have some problems. The reason may be that the attribute loss makes the data unable to be directly obtained. There is also a certain probability that the data are lost in the acquisition process, and there is also a certain probability that the object-related attributes cannot be used, and the cost of obtaining relevant data is expensive. Therefore, it is important to preprocess the original data.

Usually, data preprocessing can be conducted in the following ways, such as (1) the analysis of missing values and outliers, (2) data cleaning, (3) attribute specification, and (4) data transformation. The post demand obtained from the crawler website is regular, and there is no strong correlation between the data. Thus, it just needs to simply sort out the original data, such as the deletion of the missing value and the elimination of the same data. They are the cleaning of the data, and then, the data are misplaced.

In the process of data cleaning, it is necessary to delete the missing value in the missing row. Then, the data are deduplicated. There are two cases in this operation. One is that the eigenvalues of the data with the relevant records are completely consistent, and the other is that some eigenvalues of the data with the records are not the same. With the crawler, enterprises release the same post in different periods, and it needs to delete the repeated items. In this case, data deduplication is needed. Due to the weak correlation between the internal data, it is necessary to check the displacement and deal with the phenomenon of backward data due to after the completion of data cleaning. If the data for posts are complete, they do not need to be processed, or it needs to consider the data column. If the abovementioned process is performed, the data preprocessing is completed. In the selection of datasets, Kaggle is selected, and it is a platform proposed by Kaggle. It has a total of more than 350 datasets and 200 feature datasets. It is selected as the dataset because it can provide the needed dataset and new knowledge, as well as the applicable materials.

### Hyperparameters Setting

A neural network module is built with Python. Due to the cost of time of network training, the cost of network hardware, and others, the network structure is defined as 4-8-1 through multiple tests and related evaluation of errors. In other words, the first- to fourth-order difference data of the residual sequence are used as the input variables, and the residual sequence is used as the output variable. It is stipulated that there are eight hidden nodes and one output node. Since the learning rate of the neural network has a certain effect on the change of weights, and the extreme value of the learning rate has a bad effect on the stability of the network, the number of training is set to 1,000 times, the error target value is set to 0.000001, and other related parameters are at default state to make the network stable.

The neural network can be infinitely close to any function. The reason is that the non-linear function is added as the excitation function. After the excitation function is processed, it is transmitted to the next layer of the neural network. That is to say, the establishment of the BPNN should have an appropriate excitation function. Sigmoid in the prediction function is not easy to produce the phenomenon of gradient disappearance, and the convergence speed is relatively good. It is suitable for the post prediction of the SARIMA-BPNN and can be used as an excitation function.

### Experimental Results and Discussion

#### Comparison of Job Predictive Value and Real Value

The monthly demand data and prediction data of relevant posts of enterprises near a university in 2020 are summarized in Table 1.

**TABLE 1:** Comparison of the predictive and real posts.

Month	The real value	SARIMA-BP prediction	BP prediction
1	152	146	132
2	165	164	141
3	251	244	233
4	267	261	254
5	273	271	241
6	296	299	270
7	178	176	150
8	200	190	177
9	280	270	156
10	354	342	333
11	146	139	133
12	133	135	120

The data in Table 1 show that the RMSE calculated by the SARIMA-BP model is 7.523 by inputting the number of posts and the predicted value presented by the big data of enterprises in society into the RMSE. Also, the errors produced by the model are few, indicating that the prediction effect of the model is better and the expected value. Compared with the model based on the BPNN, the SARIMA-BP model is more excellent.

#### Trend Forecast of Post Demand

**FIGURE 7:**
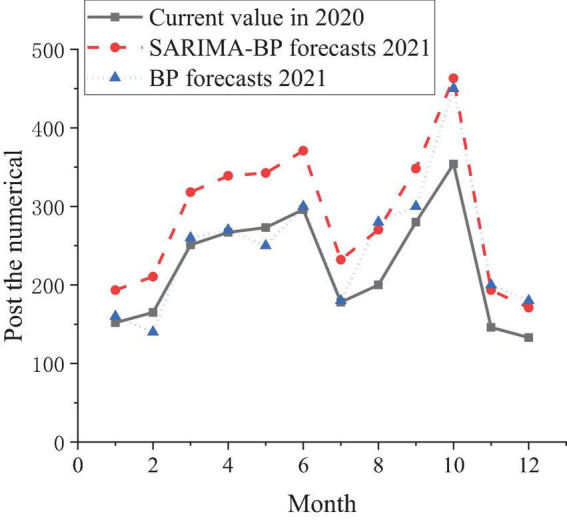
Prediction trend of job demand in 2021.

Based on the data in Table 1, the SARIMA-BP model and the model based on the BPNN are used to simulate and predict the number of posts in the next 12 months of 2021. The comparison is shown in Figure 7.

Figure 7 shows that the prediction made by the SARIMA-BP model can meet the demand for post prediction better than the model based on the BPNN, and the fitting of the model is also better. In short, colleges and governments can use the model based on the SARIMA-BPNN to analyze the matching degree of the majors opened by colleges and the posts provided by enterprises, and the talent training should be targeted for the demand of the posts provided. The “University-Industrial Research Collaboration” is proposed to help colleges and universities have a more scientific talent training strategy and cultivate more talents needed by society. In this process, prediction algorithms are used to analyze the real needs of society and provide more reliable data for colleges and universities when they formulate talent training strategies.

#### Application of Optimization Prediction Algorithm

**FIGURE 8:**
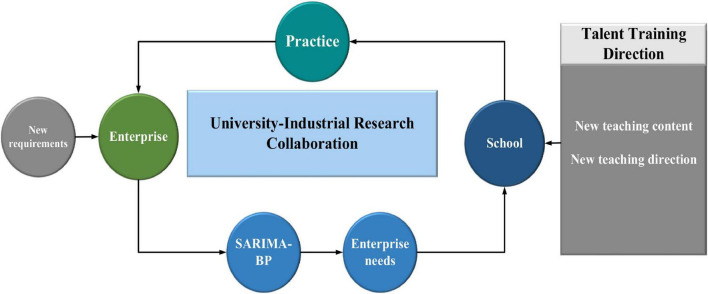
The use of the SARIMA-BP algorithm for talent training under “University-Industrial Research Collaboration”.

Figure 8 shows the strategy of using the SARIMA-BP algorithm for talent training under “University-Industrial Research Collaboration.”

With the rapid development of society, the communication design majors in colleges and universities must be cultivated to match the needs of social enterprises. The SARIMA-BP algorithm is used for talent training under “University-Industrial Research Collaboration.” The concept of “University-Industrial Research Collaboration” is implemented. In this process, the SARIMA-BP algorithm is used to predict and analyze the real needs of enterprises, so that the direction of the talent training is figured out, and a talent cultivating strategy is developed.

## Conclusion

The major, i.e., communication design, is popular nowadays. However, the talents cultivate by colleges universities mismatch the post demand by society. Under the “University-Industrial Research Collaboration,” new ideas and requirements are put forward for changing the teaching mode for talent training. Under the background of “University-Industrial Research Collaboration,” the correlation between post demand and talent training is constructed. Based on the BPNN in deep learning and the advantages of the SARIMA model, the SARIMA-BPNN model is designed after relevant parameters are adjusted to solve the problem that the error should be eliminated, and the talent demand in specific posts in social enterprises can be predicted more accurately. Through the analysis of simulation experiments, the errors produced by the designed model in the actual prediction and future prediction are reduced, which can be used in the training of communication design majors. Through this model, the posts required by society are recognized. Educational practice can provide students with better practical teaching so that the “use” of “University-Industrial Research Collaboration” can play its full role, and the enterprises, universities, research, and practice can interact with each other better. The “University-Industrial Research Collaboration” plays a great role in the cultivation of communication design talents. The shortcoming of the study is that the size of the samples is small in the discussion part, which may have a certain impact on the conclusion. In the follow-up research, more experimental data should be collected. For the training of communication design majors, the development direction suitable for society should be figured out to promote the integration of colleges and enterprises.

## Data Availability Statement

The raw data supporting the conclusions of this article will be made available by the authors, without undue reservation.

## Ethics Statement

The studies involving human participants were reviewed and approved by Anhui Agricultural University Ethics Committee. The patients/participants provided their written informed consent to participate in this study. Written informed consent was obtained from the individual(s) for the publication of any potentially identifiable images or data included in this article.

## Author Contributions

All authors listed have made a substantial, direct, and intellectual contribution to the work, and approved it for publication.

## Conflict of Interest

The authors declare that the research was conducted in the absence of any commercial or financial relationships that could be construed as a potential conflict of interest.

## Publisher’s Note

All claims expressed in this article are solely those of the authors and do not necessarily represent those of their affiliated organizations, or those of the publisher, the editors and the reviewers. Any product that may be evaluated in this article, or claim that may be made by its manufacturer, is not guaranteed or endorsed by the publisher.
